# Supplementary material for: High-field transport properties of a P-doped BaFe2As2 film on technical substrate
Source: Sci Rep. 2017 Jan 12;7:39951. doi: 10.1038/srep39951 (PMC5227693; doi:10.1038/srep39951)
Supplement: Supplementary Information [file srep39951-s1.pdf]

High-fields transport properties of a P-doped $BaFe_2As_2$ film on technical substrate

Kazumasa Iida,[1,*] Hikaru Sato,[2] Chiara Tarantini,[3] Jens Hänisch,[4] Jan Jaroszynski,[3] Hidenori Hiramatsu,[2,5] Bernhard Holzapfel,[4] and Hideo Hosono[2,5]

[1]*Department of Crystalline Materials Science, Nagoya University, Chikusa-ku, Nagoya 464-8603, Japan*

[2]*Laboratory for Materials and Structures, Institute of Innovative Research, Tokyo Institute of Technology, Mailbox R3-1, 4259 Nagatsuta-cho, Midori-ku, Yokohama 226-8503, Japan*

[3]*Applied Superconductivity Center, National High Magnetic Field Laboratory, Florida State University, Tallahassee FL 32310, USA*

[4]*Karlsruhe Institute of Technology, Institute for Technical Physics, Hermann-von-Helmholtz-Platz 1, 76344 Eggenstein-Leopoldshafen, Germany*

[5]*Materials Research Center for Element Strategy, Tokyo Institute of Technology, Mailbox SE-6, 4259 Nagatsuta-cho, Midori-ku, Yokohama 226-8503, Japan*

(Dated: October 10, 2016)

Supplementary Information

Structural characterisation

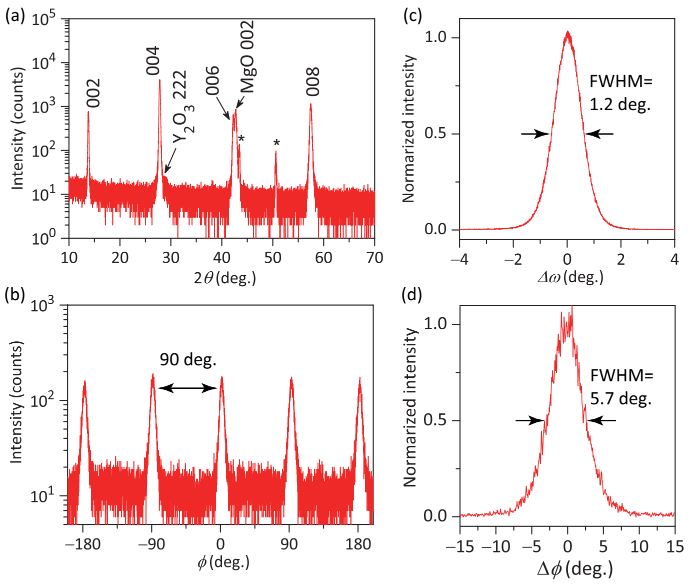

FIG. S1. **X-ray diffraction (XRD) patterns of a P-doped Ba-122 film on an IBAD-MgO substrate with in-plane misorientation angles of $\Delta\phi_{\mathrm{MgO}} = 8°$.** (a) ω-coupled 2θ scan for out-of-plane reflections. The asterisks indicate the diffraction peaks from the IBAD-MgO substrate. (b) ϕ scan of the asymmetric 103 diffraction. Intensity-normalised rocking curves of (c) the out-of-plane 004 and (d) the in-plane 200 diffraction. These XRD data indicate that (i) The planarising amorphous Y_2O_3 bed-layers in the IBAD-MgO substrate slightly crystallised as observed at $2\theta = 29°$ due to high temperature growth at 1200 °C. (ii) The P-doped Ba-122 film heteroepitaxially grew on the IBAD-MgO substrate with the orientation relation of Ba-122[001]||IBAD-MgO[001] out-of-plane and Ba-122[100]||IBAD-MgO[100] in-plane without extra in-plane rotational domains. (iii) The FWHM value of out-of-plane diffraction (i.e., tilting of crystallites) is $\Delta\omega_{\mathrm{Ba122}} = 1.2°$, which is comparable to that of the previous study [S2], whereas that ($\Delta\phi_{\mathrm{Ba122}} = 5.7°$) of in-plane diffraction (i.e., twisting of crystallites) is slightly improved from that ($\Delta\phi_{\mathrm{Ba122}} = 8°$) of the previous study mainly because of higher growth temperature employed in this study [S1, S2]. The $\Delta\phi_{\mathrm{Ba122}} = 5.7°$ is smaller than that of IBAD-MgO ($\Delta\phi_{\mathrm{MgO}} = 8°$) due probably to self epitaxy effect during PLD growth of the Ba-122 film.

Resistivity curves for determining H_{c2} and H_{irr}

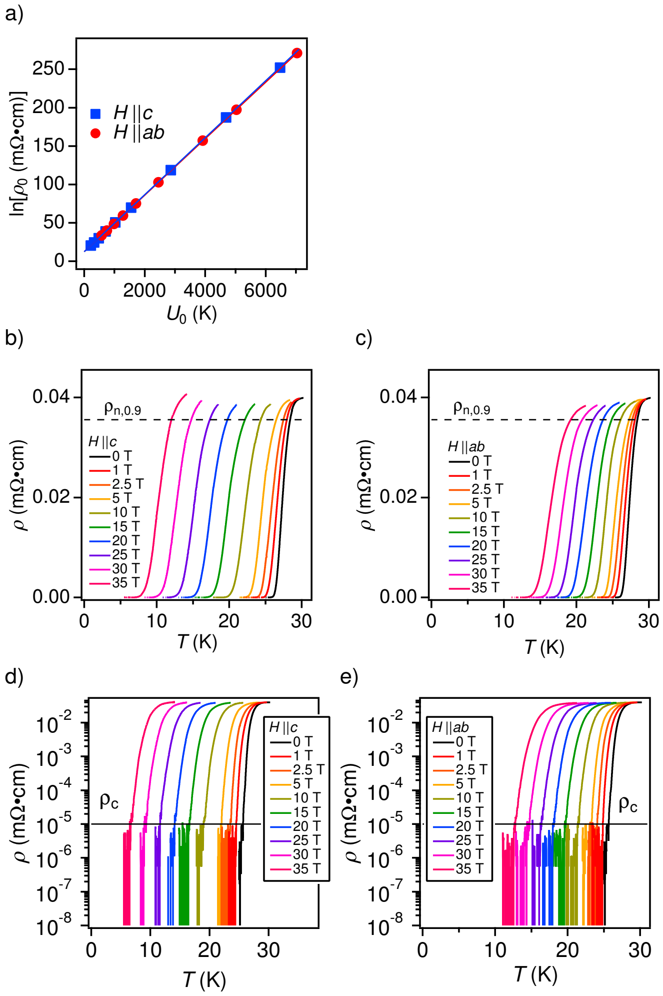

FIG. S2. **Resistivity curves for determining** H_{c2} **and** H_{irr}**:** a) Relationship between $\ln\rho_0$ and U_0 for $H \parallel c$ and $\parallel ab$. b) In-field resistivity traces $\rho(T)$ measured in static field up to 35 T for $H \parallel c$ and c) $H \parallel ab$. For determining H_{c2}, a constant resistivity criterion for which the normal state resistivity (ρ_{n}) at 28.5 K is reduced to 90% ($\rho_{\mathrm{n},0.9}$) is shown as the dotted line. d) and e) The corresponding semi-logarithmic plots. For determining H_{irr}, a resistivity criterion of $\rho_{\mathrm{c}} = E_{\mathrm{c}}/J_{\mathrm{c},100} = 1.0^{-8}\,\Omega\mathrm{cm}$ is shown. Here E_{c} is the electric field criterion ($1\,\mu\mathrm{V/cm}$) for determining J_{c} from $E - J$ measurements and $J_{\mathrm{c},100}$ is the criterion ($100\,\mathrm{A/cm^2}$) for determining H_{irr} from $J_{\mathrm{c}} - H$ measurements, respectively.

Linear presentation of $E - J$ curves at 4.2 K

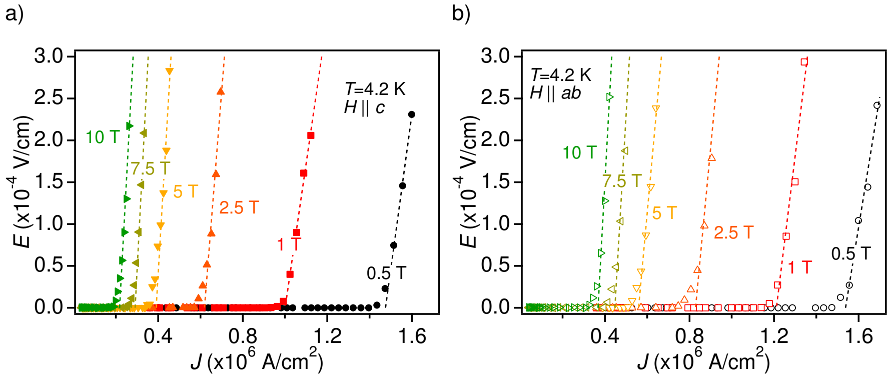

FIG. S3. $E - J$ **curves:** Linear presentation of the $E - J$ curves shown in Fig. 4 up to 10 T for a) $H \parallel c$ and b) $H \parallel ab$.

* Correspondence to: iida@nuap.nagoya-u.ac.jp

[S1] Sato, H., Hiramatsu, H., Kamiya, T. & Hosono, H. High critical-current density with less anisotropy in $BaFe_2(As,P)_2$ epitaxial films: Effect of intentionally grown c-axis vortex-pinning centers. *Appl. Phys. Lett.* **104**, 182603 (2014).

[S2] Sato, H., Hiramatsu, H., Kamiya, T. & Hosono, H. High critical-current in iron-based superconductor thin-films on metal tapes arising from large misorientation grain boundaries. *arXiv:1606.01477* , (2016).
